# Advances in 3D Printed Bone Implants: Smart Responsive Antibacterial Strategies and AI-Driven Design

**DOI:** 10.3390/biomimetics11070493

**Published:** 2026-07-14

**Authors:** Zijun Hu, Hanpeng Liu, Ding Xu, Yuan Wang, Tong Shu, Kefeng Wang, Zhiqiang Wang, Xiaofan Deng, Yuanchen Li, Ee Meng Cheng, Hao Feng, Zhaoyang Li, Caideng Yuan, Xiang Ge

**Affiliations:** 1 Key Laboratory of Mechanism Theory and Equipment Design of Ministry of Education, School of Mechanical Engineering, Tianjin University, Tianjin 300354, China; 2 Tianjin Key Laboratory of Composite and Functional Materials, School of Materials Science and Engineering, Tianjin University, Tianjin 300072, China; 3 China Automotive Parts Technology (Tianjin) Co., Ltd., Tianjin 300300, China; 4 School of Mechanical and Electrical Engineering, Nanjing University of Aeronautics and Astronautics, Nanjing 210016, China; 5 National Engineering Research Center for Biomaterials, Sichuan University, Chengdu 610064, China; 6 Tianjin Key Laboratory of High Performance Manufacturing Technology and Equipment, School of Mechanical Engineering, Tianjin University of Technology and Education, Tianjin 300222, China; 7 Tianjin Key Laboratory of Advanced Mechatronics Equipment Technology, School of Mechanical Engineering, Tiangong University, Tianjin 300387, China; 8 Faculty of Electronic Engineering & Technology, Universiti Malaysia Perlis (UniMAP), Arau 02600, Malaysia; 9 Faculty of Education and Humanities, Universiti Tun Abdul Razak, Kuala Lumpur 50250, Malaysia; 10 School of Chemical Engineering and Technology, Tianjin University, Tianjin 300350, China

**Keywords:** 3D printing, implant-associated infections, smart responsive scaffold, antibacterial strategies, artificial intelligence

## Abstract

For critical-sized bone defects, bioactive implants are indispensable. Although advanced three-dimensional (3D) printing technology enables the precise manufacturing of customized bone scaffolds, implant-associated infections (IAIs) remain a significant clinical challenge. Moreover, traditional passive antibacterial coatings often face problems such as uncontrolled release of antibacterial agents and insufficient long-term antibacterial efficacy. This review elaborates on the transformation of antibacterial strategies in the field of bone tissue engineering (BTE) from “passive” to “smart responsive” modes. We summarize the endogenous (such as pH, temperature, reactive oxygen species (ROS), and enzyme) and exogenous (such as light, microwave, and ultrasound) response systems. Notably, the ultrasound-driven strategy is highly emphasized due to its outstanding deep tissue penetrability and dual functional characteristics: it can not only eliminate stubborn biofilms through the sonodynamic effect by generating ROS, but also promote osteogenesis through the piezoelectric effect. Additionally, we also discuss the recent progress of artificial intelligence (AI) in the field of bone scaffold manufacturing. AI-driven algorithms help to rapidly optimize complex scaffold structures and accurately predict their mechanical properties, thereby effectively avoiding the inefficiencies brought about by the traditional “trial and error” method. In conclusion, combining AI-assisted manufacturing technology with smart responsive antibacterial strategies opens up a highly promising frontier field for the development of personalized and infection-free bone implants.

## 1. Introduction

Bone tissue constitutes the indispensable structural framework of the human body, providing essential mechanical support and playing an important role in movement, hematopoiesis, and the protection of vital organs [1]. As a highly mineralized tissue, bone possesses an innate regenerative capacity for minor injuries, which typically heal spontaneously via external stabilization [1]. However, severe bone defects resulting from trauma, tumors, or congenital deformities exceed the body’s intrinsic regenerative capacity. Consequently, surgical intervention and the implants are required to achieve stability [1]. Statistics indicate that millions of patients suffer annually from skeletal disorders associated with diabetes, obesity, and traumatic injuries [2]. In the United States, over 7 million orthopedic injuries are diagnosed each year, with 1.16 million patients requiring surgical intervention [3]. These defects present serious clinical challenges.

Currently, materials for repairing bone defects are broadly categorized into four types: autografts, allografts, xenografts, and synthetic bone substitutes [4]. Although autografts are considered the “gold standard”, they are limited by donor-site complications, finite supply, and anatomical mismatch [5]. While allografts and xenografts are more readily available, they carry risks associated with immunogenicity, disease transmission, and central necrosis in cases of large-scale transplantation [6]. Consequently, synthetic bone substitutes have emerged as an ideal option due to their scalability, cost-effectiveness, and availability [7]. Thus, bone tissue engineering (BTE) strategies have been developed to provide personalized structural scaffolds [8].

An ideal bone scaffold must possess a personalized architecture, balanced mechanical strength, controllable porosity, biocompatibility, and osteoinductivity [9,10,11]. Three-dimensional (3D) printing enables precise geometric control through layer-by-layer deposition, thereby facilitating the fabrication of biomimetic scaffold [12]. Furthermore, 3D printing can also create porous structures, which is vital for nutrient transport, metabolic waste removal, and the infiltration of cells [13]. Integrating 3D printing with BTE bypasses the morphological constraints of traditional manufacturing, providing a customized technological path for repairing irregular defects.

The success of BTE depends on the ability of 3D printed scaffolds to orchestrate the fundamental processes of osteogenesis. Recent advances have demonstrated that these processes are primarily mediated by three synergistic mechanisms: biomineralization, immunomodulation, and physical mechanotransduction [14,15,16]. Specifically, the controlled degradation of scaffold biomaterials establishes a localized microenvironment supersaturated with calcium and phosphate ions, which serves as a foundation for bone-like apatite deposition and structural osteoconduction [14,17]. Additionally, by actively modulating the host immune response and driving the transition of macrophages from a pro-inflammatory (M1) phenotype to a pro-healing (M2) phenotype, a favorable environment is created for the proliferation and differentiation of bone marrow mesenchymal stem cells (BMSCs), thereby promoting bone regeneration [15]. Furthermore, natural bone possesses inherent piezoelectric properties [18]. Bone scaffolds can convert physical stimuli into biophysical signals—such as local microcurrents—thereby activating voltage-gated ion channels and upregulating osteogenic pathways to accelerate the bone remodeling process [16].

**Figure 1:**
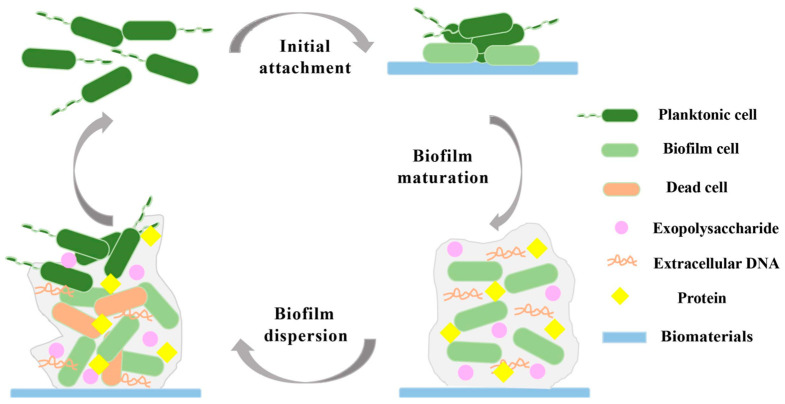
The process of biofilm formation and antibiotic resistance mechanisms. Biofilm formation is divided into three steps: Initial attachment of the biofilm; Biofilm maturation; Biofilm dispersion. Reproduced with permission from Ref. [22].

Implant-associated infections (IAIs) remain one of the most formidable complications in orthopedic surgery, severely compromising patient quality of life [19,20,21]. Bacterial colonization, primarily by Gram-negative bacteria, such as *Escherichia coli* (*E. coli*) and Gram-positive bacteria, represented by *Staphylococcus aureus* (*S. aureus*) and *Staphylococcus epidermidis* (*S. epidermidis*), leads to the rapid formation of robust biofilms (Figure 1) [22,23]. These biofilms act as physical barriers that shield bacteria from the host immune system and systemic antibiotics, significantly enhancing antimicrobial resistance (AMR) [24,25]. Current clinical reliance on systemic antibiotic administration often fails to achieve effective local drug concentrations [26]. Furthermore, the emergence of multidrug resistant superbugs, such as methicillin-resistant *Staphylococcus aureus* (MRSA), has rendered traditional antibiotic therapies increasingly inadequate [27,28].

Consequently, this review focuses on antibacterial strategies based on non-antibiotic methods. Conventionally, the clinical management of IAIs has relied on passive antibacterial strategies, such as loading inorganic antibacterial agents onto orthopedic scaffolds [7]. However, traditional systems release drugs in an uncontrolled diffusion-based manner, and they are unresponsive to the actual state of infection [29].

To address these multifaceted challenges, the development of smart responsive antibacterial materials has emerged as a transformative frontier in BTE [15]. Unlike traditional passive release systems, stimuli-responsive materials enable spatiotemporal control over the antimicrobial process, minimizing systemic toxicity while maximizing localized efficacy [29]. These strategies are broadly categorized into endogenous and exogenous responsiveness [29]. Endogenous systems capitalize on specific pathological cues within the infection microenvironment—such as localized acidification (pH) [30], hyperglycemic states (glucose) [31], or the changes in body temperature [32]—to trigger self-activating bactericidal actions. Concurrently, exogenous stimuli, including light [33], ultrasound [34], and microwave irradiation [35], provide a non-invasive means of controlled therapy, leveraging physical effects to dismantle biofilms in deep-seated tissues [36].

Furthermore, driven by the escalating clinical demand for patients’ specific implants that traditional trial-error approaches can no longer fulfill, BTE is undergoing a profound paradigm shift toward artificial intelligence (AI)-assisted 3D scaffold design [37]. By leveraging advanced machine learning (ML) algorithms to analyze high-dimensional biomechanical data, AI-driven frameworks can precisely optimize multiscale porous architectures, while concurrently predicting long-term osseointegration and mechanical performance [38]. Therefore, integrating these AI algorithms with advanced 3D printing technologies not only enhances manufacturing precision but also accelerates the transition from research to application, thereby fostering the advancement of BTE.

This review comprehensively summarizes recent progress in 3D printed scaffolds for bone regeneration. Specifically, we systematically evaluate advanced 3D printing techniques, material selection, and key manufacturing parameters, which establish a structural foundation for the fabrication of bone scaffolds. We also dissect the mechanisms of passive and smart-responsive antibacterial strategies, exploring endogenous and exogenous stimuli response approaches. Then, we analyze how to achieve synergistic effects between infection eradication and osteogenesis. Finally, we present how AI algorithms can optimize scaffold structures and predict their performance, and we offer a promising perspective on the potential applications of AI-assisted design.

## 2. Manufacturing of 3D Printed Scaffolds

### 2.1. Advanced 3D Printing Technologies

Recently, 3D printing technology is being used more and more frequently in the fabrication process of orthopedic implants [39]. Additive manufacturing can combine advanced biomaterials with biomimetic structures, and accurately create bone defect repair scaffolds that meet the specific conditions of patients [40]. These scaffolds are made based on the digital anatomical data of the patient’s bone defects. Generally, the production of such implantable devices follows a three-step process: radiological data acquisition of the defect, 3D digital reconstruction, and subsequent additive manufacturing [41]. The clinical and research areas are mainly dominated by three printing modes, namely extrusion printing, photopolymerization printing, and powder printing [39], each with different spatial resolution and scaling capabilities. This section reviews the 3D printing technologies used for manufacturing bone scaffolds.

#### 2.1.1. Extrusion

**Figure 2:**
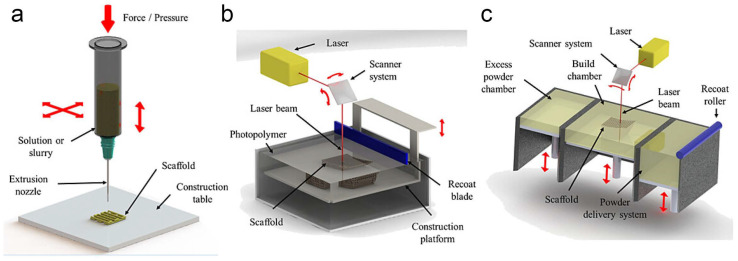
A brief schematic of different printing methods. (**a**) Extrusion-based techniques; (**b**) Photopolymerization techniques; (**c**) Powder bed fusion. Reproduced with permission from Ref. [57].

Extrusion-based additive manufacturing technologies—including fused deposition modeling (FDM) and direct ink writing (DIW)—operate by extruding material inks through a nozzle under the influence of pneumatic or mechanical pressure (Figure 2a) [42]. FDM primarily processes thermoplastic polymers, such as polylactic acid (PLA), polycaprolactone (PCL), and polyether ether ketone (PEEK), which functions by melting filaments through heating and depositing them layer-by-layer onto a platform [43,44,45,46]. In contrast, DIW operates at ambient temperature, functioning by extruding high viscosity bio-inks. These materials subsequently undergo post-processing steps—such as ionic crosslinking or curing—to achieve structural stabilization [47,48]. Although the resolution of DIW is typically lower than other high-precision printing methods, its operation under mild conditions offers distinct advantages, enabling the preservation of the bioactivity of heat-labile biomolecules and the viability of osteoblasts [49]. Furthermore, DIW accommodates a series of materials, ranging from bioceramics and hydrogels to composite mixtures, thereby highlighting its exceptional material versatility [49]. When combined with its comparatively low equipment and operational costs, these attributes have solidified the position of DIW as a foundational and widely adopted technology in the field of BTE [49,50].

#### 2.1.2. Photopolymerization

Photopolymerization is one of the fundamental modes in the field of additive manufacturing, including digital light processing (DLP) and stereolithography (SLA) [51]. This type of technology uses precisely controllable optical wave—spanning infrared (IR), ultraviolet (UV), or visible light wavelengths and can be built layer by layer to enable spatially selective crosslinking reactions of photosensitive bioink as shown in Figure 2b [51]. And it also has clear advantages, not only can it achieve micrometer level feature resolution, but the surface is also particularly smooth [49]. The printing technology based on photopolymerization is perfectly suited for manufacturing complex geometric components, and the resulting structures have good fidelity. In BTE related applications, the morphology of the scaffold surface can greatly affect cell behavior and nutrient transport, which is a critical requirement [49].

Zhang et al. developed a magnetic responsive multifunctional scaffold using cryogenic vat photopolymerization (cryo-VPP), wherein UV-induced crosslinking was synergistically combined with an external magnetic field to precisely help regenerate auricular cartilage and reduce local inflammatory reactions [52]. Wu et al. used DLP technology to replicate the spiral architecture of natural conch shells, fabricating β-tricalcium phosphate (β-TCP) scaffolds that guide cell migration and promote distal bone tissue regeneration [53]. Collectively, photopolymerization can not only produce high-resolution and structurally complex structures, but also add spatially limited functional clues to help with tissue repair.

#### 2.1.3. Powder Bed Fusion

Powder bed fusion printing technologies, including selective laser sintering (SLS) and electron beam melting (EBM), utilize high-energy beams to selectively fuse powdered materials (Figure 2c) [54]. In SLS, a laser source scans the powder bed to induce localized sintering or partial melting; conversely, in EBM, an electron beam is employed under vacuum conditions to achieve the complete melting and consolidation of metal powders [55]. In the context of fabricating ceramic scaffolds, SLS is generally preferred over EBM, as it facilitates partial sintering rather than complete melting—a characteristic that helps preserve the material’s inherent bioactivity and porous structure, both of which are critical for osseointegration [56]. Qin et al. reported that the porous calcium tetraphosphate scaffold fabricated by SLS induced the rapid self-assembly of a bone-like apatite layer on their surfaces after being immersed in simulated body fluid (SBF) for just 3 d [56]. Furthermore, Sheng et al. utilized EBM to construct porous titanium (Ti) alloy scaffolds functionalized with dual-ion crosslinked hydrogels [55]. This integrated system enabled spatiotemporal control over immune-mediated osteogenesis, promoting the polarization of M2-type macrophages while inhibiting osteoclast genesis in an osteoporosis model [55]. Collectively, these studies highlight the versatility of powder-based printing approaches in generating metal or ceramic scaffolds.

### 2.2. Materials Selection

Biomaterial-based scaffolds play a vital role in mimicking the extracellular matrix while simultaneously serving as delivery systems for bioactive cells and molecule [58]. Consequently, the selection of biomaterials—broadly categorized into metals, bioceramics, and biopolymers—is critical for achieving a balance among mechanical strength, biodegradability, and antibacterial activity.

#### 2.2.1. Metals

Metals and their alloys remain the core materials for bone scaffolds due to their superior mechanical strength, excellent fatigue resistance, and long-term structural durability [54]. Among these, Ti and its alloys (e.g., Ti6Al4V) are the most widely utilized permanent orthopedic implants in clinical practice [59]. 3D printing technologies—such as SLS and EBM—enable the precise fabrication of porous Ti scaffolds featuring mathematically defined structures (e.g., triply periodic minimal surface (TPMS) designs) [60]. This topological optimization significantly reduces the elastic modulus of Ti, bringing it into close alignment with that of human bone; this effectively mitigates stress shielding while simultaneously maintaining a highly interconnected porous network to promote osteogenesis and vascularization [60]. However, the inherent bio-inertness and lack of antibacterial activity characteristic of Ti-based scaffolds often necessitate secondary surface functionalization treatments to prevent early-stage IAIs [61,62].

#### 2.2.2. Bioceramics

Bioceramics play a vital role in BTE due to their exceptionally high mechanical stiffness and remarkable osteoconductivity [63,64]. Among these, calcium phosphate-based ceramics—particularly hydroxyapatite (HA) and tricalcium phosphate (TCP)—are the most extensively studied types [63]. HA exhibits excellent osseointegration properties and provides the necessary active sites for osteoblast attachment and subsequent biomineralization; however, its slow degradation rate *in vivo* often limits rapid tissue replacement [65]. Conversely, α-TCP and β-TCP possess significantly faster degradation rates [66]. Consequently, blending HA and TCP to form biphasic calcium phosphate (BCP) allows for the precise tuning of the degradation rate to match that of nascent bone [2]. In the context of 3D printing, incorporating nano-HA or TCP into a printable polymer matrix (such as PCL) remains a common strategy. This composite approach significantly enhances the mechanical strength of the scaffold while maintaining excellent printability and promoting *in vivo* osteoinductive capabilities [46]. Beyond traditional calcium phosphates, bioactive glasses (BGs) have drawn considerable attention [67,68]. BGs not only possess osteogenic potential but also release specific dissolution products, such as soluble silicate and calcium ions—that continuously upregulate the expression of osteogenic and angiogenic genes [69].

#### 2.2.3. Biopolymers

Biopolymers, including natural and synthetic polymers, are extensively utilized in 3D printed bone scaffolds due to their exceptional biocompatibility, tunable biodegradability, and structural versatility [70,71]. Natural polymers inherently mimic the extracellular matrix (ECM), providing a highly hydrated environment conducive to cell proliferation [72]. For instance, multi-component hydrogel inks formulated with sodium alginate (SA), carboxymethyl chitosan (CMCS), and gelatin exhibit excellent rheological shear-thinning properties, making them highly suitable for extrusion-based 3D bioprinting [73,74,75].

**Figure 3:**
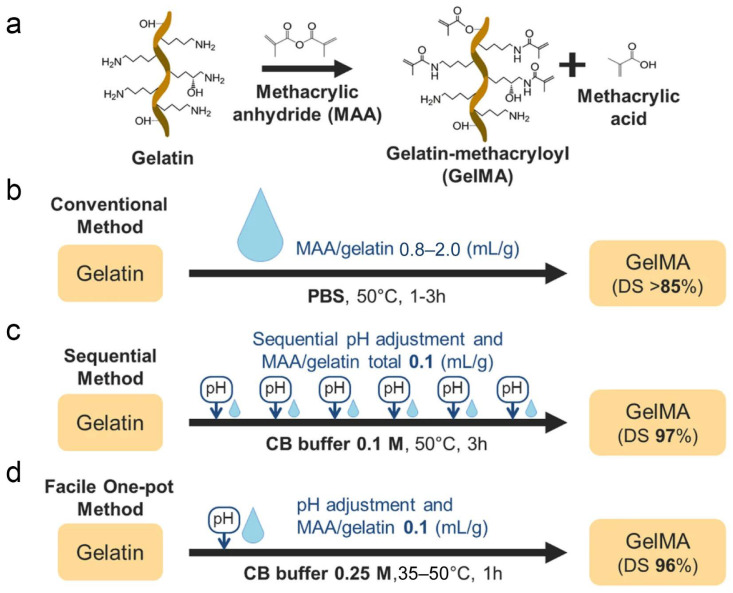
(**a**) Schematic illustration of GelMA synthesis; (**b**) Conventional method for GelMA synthesis; (**c**) Sequential method for GelMA synthesis; (**d**) Facile one-pot method for GelMA synthesis. PBS: Phosphate-buffered saline; DS: Degree of substitution; CB: Carbonate-bicarbonate. Reproduced with permission from Ref. [79].

Among natural biopolymers, gelatin methacryloyl (GelMA) has garnered tremendous attention due to its highly ECM-like properties, excellent photocrosslinking capability, and superior biocompatibility [76,77]. The method of synthesizing GelMA was originally developed by Van Den Bulcke et al. [78]. Briefly, methacrylic anhydride (MAA) monomers were reacted with lysine and hydroxyl lysine groups of gelatins (Figure 3a) by dissolving gelatin in phosphate-buffered saline (PBS) solution at 50 °C (Figure 3b) [79]. The conventional method requires a large amount of MAA [79]. Although sequential processing reduces MAA consumption, multiple pH adjustments are needed, being followed by MAA addition (Figure 3c) [79]. Shirahama. et al. proposed the facile one-pot synthesis method, minimized both MAA consumption and manual work (Figure 3d) [79].

GelMA serves as an ideal structural bioink capable of encapsulating living cells (e.g., bone marrow stem cells) while maintaining exceptional cell viability [76]. However, since pure GelMA lacks inherent osteogenic activity, it is frequently hybridized with bioactive components, such as platelet-rich plasma (PRP) or inorganic nanofillers [80]. These GelMA-based composite scaffolds provide a conducive microenvironment for osteogenesis [80]. In a recent study, Wang et al. developed a novel bioink comprising GelMA, alginate methacrylate, and HA to print highly intricate bone ECM scaffolds, which supported long-term cultivation and progressive maturation of large-scale bone organoids, promoted multicellular differentiation, and provided insights into early bone formation [81]. The intrinsic self-mineralizing property of this bioink is closely similar to natural bone, enabling bone repair both *in vitro* and *in vivo* [81].

Biopolymers can also be engineered to serve as a first line of defense against IAIs. For instance, incorporating quaternized chitosan (HACC) into natural hydrogel networks endows the scaffold with intrinsic, passive antimicrobial properties by electrostatically disrupting bacterial cell membranes [82]. Synthetic polymers serve to compensate the inherent mechanical limitations of natural hydrogels. Robust synthetic polymers, such as poly(lactic-co-glycolic acid) (PLGA), PEEK, and PCL are frequently employed to provide macroscopic mechanical support [46,83].

Below is Table 1 illustrating the comparison of advanced 3D printing technologies for bone scaffold fabrication.

### 2.3. Structural Design of Scaffolds

The structural design of 3D printed bone scaffolds is of paramount importance [84]. However, a dilemma arises regarding the optimization of these structural features: the very characteristics that promote osteogenesis also tend to facilitate bacterial adhesion [84]. This section explores this contradiction through the porosity and microtopography, analyzing how these parameters exert distinct effects on osteoblasts and bacteria, respectively.

#### 2.3.1. Porosity and Pore Sizes

In BTE, a highly interconnected porous network is essential to facilitate cell migration, nutrient diffusion, and neovascularization [85,86,87]. It is generally accepted that a minimum pore size of 300 µm, coupled with an overall porosity ranging from 60% to 80%, constitutes the optimal condition for promoting rapid bone growth and osseointegration [54,88]. Typically, pore sizes of ≥300 µm are requisite for inducing new bone formation and vascularization, whereas pore sizes of <100 µm may severely inhibit angiogenesis [89]. Considering the balance between bone scaffold porosity and mechanical strength, an average pore size of approximately 500 µm has been widely validated as the optimal threshold for promoting bone regeneration [90].

However, these interconnected internal pores significantly increase the surface area, thereby creating a favorable environment for bacterial attachment. More critically, these deep pores physically shield bacteria from the effects of fluid shear forces [91]. This substantially elevates the risk of IAIs [91]. Consequently, relying solely on the optimization of microporous parameters is insufficient; structural design must carefully balance osteoconductive permeability with mechanical stability, while inevitably necessitating the integration of antibacterial strategies to mitigate the infection risks inherent in highly porous networks.

#### 2.3.2. Topological Surface Features

In the context of osteogenesis, surface roughness plays a powerful role in promoting osseointegration. The presence of microscopic surface protrusions not only increases the specific surface area available for targeted protein adsorption but also significantly enhances osteoblast adhesion, spreading, and the subsequent process of ECM mineralization [61,92]. However, this high degree of surface roughness simultaneously provides an ideal physical sanctuary for bacterial colonization, shielding bacteria from external shear forces [92].

Simply polishing the implant surface is not a viable solution [93]. Ivanova et al. demonstrated that even Ti films with atomically smooth surfaces remain susceptible to colonization by *S. aureus* [93]. Furthermore, the inherent wettability of the substrate material dictates its affinity for specific bacterial species. For instance, hydrophobic polymeric surfaces (e.g., unmodified PCL or PLA) are readily colonized by hydrophobic bacterial strains, such as *S. epidermidis* [94].

Consequently, relying solely on the random topological structures that naturally emerge during the 3D printing process is fundamentally insufficient and often detrimental to infection prevention. To fully capitalize on the benefits of osseointegration, it is imperative to transition from random, rough surfaces to meticulously engineered surface topographies.

In previous studies, Ge et al. demonstrated that the rational modulation of surface topography can profoundly influence the fate of bacteria [95,96]. For example, the precise design and control of periodic arrays of micropillars can significantly reduce bacterial adhesion, curtail proliferation, and physically impede the subsequent formation of biofilms [95,96]. Building upon this foundation, Zhu et al. utilized two-photon polymerization 3D printing technology to directly fabricate shark-skin-like micro/nanoarrays [97]. This precisely engineered topography achieved remarkable non-chemical antibacterial effects, successfully inhibiting the proliferation of pathogenic bacteria by over 80% within 24 h while maintaining excellent biocompatibility [97].

Therefore, the rational and precise design of the micro/nanostructures of scaffolds serves as the cornerstone for developing the next generation of intrinsically antibacterial bone implants.

## 3. Fundamental Mechanisms of Antibacterial Action

Understanding the fundamental mechanisms of antibacterial action is crucial for treating IAIs. Pathogenic infections in bone tissue typically manifest in two distinct states, planktonic bacteria and biofilms—which require completely different antibacterial strategies [29]. The following section will discuss the antibacterial mechanisms against planktonic bacteria and biofilms in detail.

### 3.1. Planktonic Bacteria

Planktonic bacteria exist as free-floating, independent cells. In this state, cell walls and membranes of planktonic bacteria are directly exposed to the microenvironment, making them highly vulnerable to contact-killing and intracellular interference.

#### 3.1.1. Metabolic Interference

The sustained release of antibacterial metallic ions from the scaffold facilitates their penetration through the bacterial cell wall [69]. Inside the cytoplasm, these ions bind tightly to thiol-containing enzymes, critically interfering with respiratory chains, altering enzyme configurations, and ultimately causing metabolic collapse [98].

The explosive generation of reactive oxygen species (ROS) is also a major antibacterial mechanism [99]. Highly active ROS directly induce lipid peroxidation of the exposed bacterial cell membrane, leading to a loss of structural integrity. Concurrently, ROS penetrate the cellular envelope to inflict oxidative damage upon critical biomacromolecules, resulting in the cleavage of genomic deoxyribonucleic acid (DNA) and the irreversible denaturation of essential metabolic proteins [100].

#### 3.1.2. Membrane Disruption

Direct disruption of the cellular boundary provides a rapid pathway for bacterial inactivation. Chemically, cationic polymers (e.g., HACC) utilize positive charges to electrostatically bind and penetrate the negatively charged bacterial membrane, causing fatal cytoplasmic leakage [101]. Physically, engineered micro/nano-topographies—such as highly ordered TiO_2_ nanotubes—can mechanically stretch, pierce, and rupture bacterial membranes upon direct contact [95,96].

### 3.2. Biofilm

Once pathogens colonize an implant surface, they rapidly undergo a phenotypic shift to form biofilms [22]. Biofilms are structured communities of bacterial cells encased in a dense, self-produced extracellular polymeric substance (EPS) matrix [24]. This matrix creates a robust physical barrier that significantly restricts the penetration of systemic antibiotics and host immune cells, rendering the bacteria within the biofilm inherently tolerant and resistant to treatment [24,25,102]. Consequently, the primary mechanisms for eliminating biofilms can be summarized by a single strategy: disrupting the matrix and reverting the bacteria to a planktonic state.

By degrading extracellular polysaccharides and disrupting the adhesive EPS matrix, the protective layer is removed, forcing dormant bacteria within it to transition back to a planktonic form [103]. This critical structural disruption can be achieved through biochemical means, such as using matrix-degrading enzymes to cleave the adhesive components of the EPS, or via biophysical stimuli like ultrasonic cavitation, which generates intense mechanical shear forces and microfluidic effects to physically tear the biofilm apart [103,104]. Once the EPS barrier is breached and the bacteria return to a planktonic state, they lose their collective resistance and become highly susceptible to the previously described mechanisms of membrane disruption, and metabolic interference, ultimately leading to the complete eradication of the biofilm.

## 4. Passive Antibacterial Strategies

Currently, systemic antibiotic treatment and surgical removal of infected tissues are the main approaches for treating IAIs [102]. Due to the emergence of multi-drug-resistant bacteria, the efficacy of traditional therapies has significantly declined [27,28]. Non-antibiotic antibacterial strategies are increasingly gaining attention in modern orthopedics [105]. Among them, the passive antibacterial strategy—that is, mainly utilizing the inherent physical and chemical properties of biomaterials for sterilization—has become an important means for continuously protecting implants from bacterial colonization.

### 4.1. Metal or Metallic Cation

Adding broad-spectrum antibacterial metal nanoparticles (NPs) [69] and their corresponding ions to 3D printed scaffolds is currently a common passive antibacterial strategy [106,107,108]. Unlike traditional antibiotics, metal-based antibacterial agents employ multiple mechanisms to combat pathogens, such as disrupting the cell membrane, causing protein denaturation, and generating ROS [98]. This characteristic significantly avoids the emergence of bacterial resistance.

**Figure 4:**
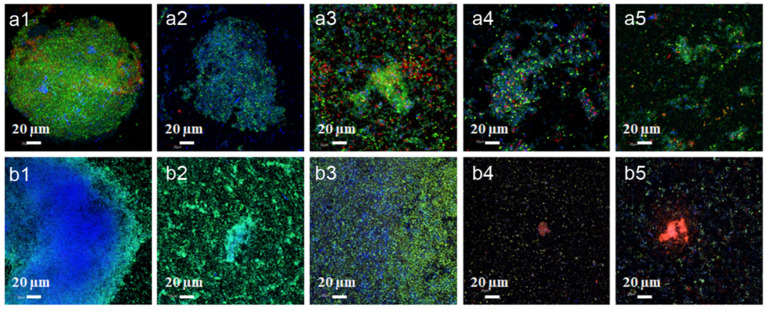
Confocal images of (**a**) *E. coli*; and (**b**) *S. aureus* biofilm degradation after 24 h in contact through a transwell with mesoporous bioactive glass scaffolds with different concentrations of AgNPs. (**a1**,**b1**) Control group; and samples containing (**a2**,**b2**) 0%; (**a3**,**b3**) 0.15%; (**a4**,**b4**) 0.3%; and (**a5**,**b5**) 1% AgNPs concentration, respectively. Controls correspond to biofilms incubated in the absence of scaffolds. Live bacteria were stained in green, dead bacteria in red, and the protective extracellular polysaccharide matrix biofilm in blue. Reproduced with permission from Ref. [69].

Silver (Ag), as a broad-spectrum antibacterial agent, possesses outstanding germicidal capabilities [107]. By embedding metallic AgNPs directly into 3D printed mesoporous bioactive glass scaffolds, Sandra et al. have achieved sustained, localized release of Ag^+^, demonstrating that bacterial growth inhibition and biofilm destruction are directly proportional to the AgNPs concentration (Figure 4) without compromising the morphology of preosteoblastic cells [69]. To further mitigate the potential burst-release toxicity and agglomeration of bare AgNPs, advanced synergistic co-dispersing NPs have been engineered. For instance, Shuai et al. incorporated a graphene oxide (GO)-AgNPs into 3D printed polymeric matrices, which leverages the physical “capturing” capacity of GO nanosheets and the “killing” effect of intercalated AgNPs, achieving an extraordinary bacterial inhibition rate of over 95% against both Gram-positive and Gram-negative strains [109]. Moreover, multi-metallic platforms, such as Ag/Zinc (Zn)-coated black phosphorus (BP@(Zn+Ag)) nanocomposites embedded in 3D printed nanofibrous scaffolds, exhibit excellent synergistic antibacterial activity while significantly facilitating osteoblast differentiation [106].

Although Ag-loaded biomaterials are widely studied, the intimate contact between Ag-coated implants and native bone raises concerns regarding the potentially harmful accumulation of Ag^+^ in surrounding soft tissues [110]. To address this issue, other metal dopants have been discovered by researchers, such as copper (Cu) and zinc (Zn), which can actively participate in osteogenic processes and angiogenesis [111]. Cu exhibits broad-spectrum antibacterial activity, including effectiveness against drug-resistant bacteria such as MRSA [112]. In the meantime, Cu can accelerate wound healing by promoting endothelial cell proliferation and angiogenesis, while also playing a crucial role in bone metabolism and bone tissue growth [111]. Pillai et al. used extrusion 3D printing technology to fabricate calcium phosphate bone cement (CPC) scaffolds loaded with different concentrations of Cu nanoparticles [113]. They found that the CPC-Cu scaffold with 1 wt% Cu NPs could significantly promote the proliferation of human bone marrow mesenchymal stem cells, increase alkaline phosphatase activity and angiogenic potential *in vitro*, and exhibit concentration-dependent antibacterial activity against *S. aureus* [113]. Thus, it comprehensively enhanced the osteogenic, angiogenic and antibacterial properties of the scaffold.

Zn is another element that has been proven to have excellent antibacterial properties. Chen et al. incorporated Zn + Ag nanocomposite materials into the PLLA scaffold. The released Zn^2+^ not only effectively inhibits bacterial growth, but also promotes the mineralization process and the proliferation of osteoblasts by facilitating the synthesis reactions in bone metabolism [106]. Bejarano et al. constructed a BG scaffold doped with Cu/Zn, which significantly enhanced the scaffold’s ability to resist methicillin-resistant MRSA [111]. More importantly, this strategy achieved a dual synergistic effect by using Cu to promote angiogenesis and Zn to accelerate the osteogenic differentiation process [111].

Gallium (Ga) is expected to become a highly promising antibacterial agent, and its excellent antibacterial properties stem from its position in the periodic table [114,115]. As a post-transition metal belonging to both the 13th group and the 11/12th group, Ga has a similar atomic structure to Cu/Zn, which enables Ga to interfere with the trivalent iron ions (Fe^3+^) in the bacterial metabolic process, thereby blocking the bacteria’s uptake of Fe elements [114,115]. The unique antibacterial mechanism of Ga avoids the problem of cytotoxicity caused by other metal antibacterial agents [114]. Wang et al. prepared a PCL scaffold containing Ga [116]. *In vitro* experiments demonstrated that the scaffold had antibacterial effects against MRSA and *E. coli* [116]. The scaffold also significantly inhibited osteoclast activity and promoted osteogenic differentiation, indicating that Ga has excellent bone healing ability [116]. Shi et al. developed a dual-functional composite scaffold, which was constructed by Ga, chitosan, silk fibroin, and umbilical cord mesenchymal stem cell exosomes; among them, Ga^3+^ is responsible for providing antibacterial activity, while the exosomes are responsible for continuous release to promote angiogenesis. In the diabetes wound model, this scaffold exhibited a synergistic effect of antibacterial, anti-inflammatory, and promoting angiogenesis, significantly accelerating the wound healing process [117].

### 4.2. Metallic Oxide

Zinc oxide (ZnO), as an inorganic antibacterial material with great application prospects, also possesses excellent biocompatibility [118]. Previous studies have confirmed that ZnO nanostructures can exhibit broad-spectrum antibacterial activity through the continuous release of Zn^2+^ ions [119]. It is worth noting that as an indispensable trace element in the human body, Zn^2+^ plays an important role in various biological processes, including maintaining enzyme structure and the integrity of DNA; this endows the antibacterial system based on ZnO with good biological safety [120]. Chen et al. demonstrated that by modifying a 3D printed composite scaffold (barium titanate (BaTiO_3_)/HA) with ZnO, a highly promising dual-functional clinical solution could be constructed, which integrates broad-spectrum antibacterial efficacy and strong bone-inducing properties [121].

Ti dioxide (TiO_2_) is another widely popular antibacterial material whose passive antibacterial capacity is limited in the dark [122]. To enhance its antibacterial effect, researchers have utilized its photocatalytic properties, enabling it to generate a large amount of ROS when exposed to light [123,124]. Broad-spectrum Gram-negative and Gram-positive bacteria can all be killed by TiO_2_ activated by light [123]. For instance, Yang et al. successfully constructed a TiO_2_ superstructure on the surface of Ti alloy implants, which not only achieved strong near-infrared (NIR) responsive antibacterial activity but also enhanced the adhesion ability of human gingival fibroblasts [125].

### 4.3. Organic Agents

Organic antibacterial agents primarily comprise three major categories: chitosan, chitosan derivatives, and antimicrobial peptides (AMPs) [126,127]. Chitosan is a naturally occurring cationic polysaccharide that has garnered significant attention in the field of antibacterial materials due to its broad-spectrum antimicrobial activity, excellent biocompatibility, and biodegradability [82,128]. Its antibacterial mechanism is primarily attributed to electrostatic interactions between the protonated amino groups and the negatively charged bacterial cell membrane, leading to increased membrane permeability, leakage of intracellular constituents, and subsequent bacterial death [128]. With the rapid development of additive manufacturing, chitosan-based antibacterial hydrogels can be precisely fabricated into high-fidelity, multi-scale porous scaffolds via 3D printing [129,130]. Notably, the coordination of aluminum (Al) ions has been utilized to enhance the printability of high-viscosity chitosan/acrylamide inks while preserving the scaffold’s antibacterial characteristics and pro-healing functions [131].

To further enhance its antibacterial efficacy and expand its application scope, various physical, chemical, and biological modification strategies have been employed to functionalize chitosan [128]. As broad-spectrum cationic biocides, HACC leverages its positive charge to electrostatically interact with and penetrate negatively charged bacterial membranes, exerting potent inhibitory effects against a diverse array of pathogens [101]. Based on this passive contact-killing foundation, Ma et al. developed an injectable photo-crosslinking hydrogel composed of HACC, methacrylate-grafted hyaluronic acid, and two-dimensional transition metal carbides, nitrides, or carbonitrides (MXene) [82]. This system combines inherent antibacterial properties with photothermal synergistic bactericidal effects which presents significant potential for the treatment of infected skin wounds [82].

AMPs are derived from innate host defensive systems. Although their antibacterial efficiency may be relatively lower than that of conventional antibiotics, they can effectively eradicate bacteria without bacterial resistance [105]. Bacteria can be killed by AMPs which form pore-forming structures within the bacterial membrane, leading to a loss of membrane integrity [127]. Antibacterial enzymes function as vital components by degrading critical bacterial structures and actively disrupting the formation of bacterial biofilms [105].

Consequently, the application of AMPs, enzymes, and other functional proteins exhibits promising clinical potential in the IAIs [132].

Below (Table 2) is the summary of passive antibacterial strategies.

## 5. Smart Responsive Antibacterial Strategies

Despite their clinical efficacy, passive antibacterial strategies face inherent limitations regarding precise dose control, long-term release kinetics, and potential cytotoxicity [133]. To overcome these limitations, smart responsive antibacterial strategies have been developed, thus achieving precise treatment.

### 5.1. Endogenous Antibacterial Strategies

Bacterial infections manifest as complex polymicrobial systems residing within a highly distinct metabolic microenvironment. This pathological niche is intrinsically characterized by acidic conditions, localized hyperthermia, high levels of ROS, and the secretion of specific extracellular enzymes [134,135]. Endogenous stimuli-responsive smart materials leverage these unique microenvironmental cues as triggers. Upon direct contact with these internal stimuli, the materials undergo programmed structural or physical-chemical alterations to execute the on-demand release of antibacterial factors [136]. This section outlines the endogenous stimulus-responsive antibacterial strategies, including pH-responsive, temperature-responsive, ROS-responsive, and enzyme-responsive antibacterial strategies.

#### 5.1.1. pH-Responsive Strategies

In the context of IAIs, the anaerobic fermentation of pathogenic bacteria and the subsequent localized inflammatory response invariably induce a pronounced acidic microenvironment with local pH values typically dropping from 7.4 to a range of 4.5–6.5 [137]. Leveraging this phenomenon, pH-sensitive polymers can be utilized as carriers to design pH-responsive antimicrobial materials [30]. pH-sensitive scaffolds or surface coatings are engineered by incorporating proton-releasing functional groups (such as amine or imidazole residues) or acid-labile chemical linkages (such as imine, hydrazone, or ester bonds) [138]. These materials maintain structural integrity and prevent antimicrobial release under normal physiological conditions (pH ~7.4) [139]. Upon exposure to the acidic microenvironment of infected tissues, the polymers undergo protonation or hydrolysis, leading to structural disintegration and controlled release of antimicrobial agents for targeted antibacterial action [139].

pH responsive approaches can allow a selective release approach either through changing the solubility of the drug carrier or through cleaving of pH responsive bonds upon variation in microenvironment pH [137,140]. Based on this distinct discovery, researchers have built various 3D nanocomposite scaffolds that release antibacterial drugs on demand when triggered by acid [141]. Ayşe Karakeçili developed a vancomycin-loaded zeolitic imidazolate framework-8 (ZIF-8) nanocrystal/chitosan composite scaffold that fully exploited the pH-responsive dissolution of ZIF-8 under acidic conditions, achieving rapid and sustained vancomycin release in a simulated bone infection inflammatory environment (pH 5.4), thereby significantly enhancing antibacterial activity against *S*. *aureus* while maintaining favorable osteogenic potential [141]. Cicuéndez et al. developed hierarchical mesoporous-microporous 3D nanocomposite scaffolds loaded with levofloxacin [142]. They revealed that the acidification of the microenvironment alters the interaction rate between the drug molecules and the mesoporous silica matrix, prompting a massive localized release of the therapeutic agent at the infection site [142]. Lin et al. developed a 3D printed chitosan composite scaffold that achieved pH-responsive therapy within the acidic microenvironment of osteomyelitis [143]. They loaded vancomycin-carrying microspheres onto the scaffold via acid-degradable Schiff base linkages [143]. This design enabled the targeted, on-demand release of vancomycin, thereby efficiently eradicating *S. aureus* [143]. More importantly, while demonstrating potent antibacterial efficacy, these pH-responsive scaffolds also exhibited excellent osteoconductivity and biocompatibility [143].

#### 5.1.2. Temperature-Responsive Strategies

The inflammatory response triggered by bacterial infection always leads to local high fever, causing a significant increase in the temperature of the infected tissue (39–42 °C) [137]. This local heat phenomenon has inspired another endogenous trigger factor for intelligent antibacterial therapy [144].

The temperature-responsive system mainly utilizes polymers with a lower critical solution temperature (LCST) characteristic, among which poly(N-isopropylacrylamide) (PNIPAM) and its derivatives are the most extensively studied materials [145]. By precisely regulating the LCST of the polymer matrix to be slightly higher than normal body temperature but lower than the high temperature caused by the inflammation, these scaffolds can achieve excellent targeted release effects [145]. The methods to achieve this goal include changing the ratio of hydrophobic components to hydrophilic components, or modifying the end groups of the polymer [146]. In the local high-temperature environment at the infected implant site, this polymer undergoes a drastic phase transition from hydrophilic to hydrophobic [147]. This transition leads to rapid and intense changes in its 3D network structure, causing the volume of the scaffold to shrink sharply, and the bactericidal agents encapsulated in the matrix are squeezed out, thereby achieving drug release at the infected site [147]. In addition to single stimulus response, Gao et al. also developed a dual-responsive polyurethane (PU) material, which ingeniously combines the temperature-sensitive LCST characteristic with pH-triggered bactericidal activity [32]. This synergy can switch the scaffold from passive antibacterial action to active responsive bactericidal mode without any external intervention.

#### 5.1.3. ROS-Responsive Strategies

IAIs represent a typical highly oxidative stress microenvironment [148]. When bacteria invade the surrounding tissues of the bone, the host’s immune cells will secrete a large amount of endogenous ROS to combat the pathogens [149]. At the same time, the bacterial metabolism will also produce certain ROS [148]. Sensitive chemical bonds of ROS can be introduced into the intelligent responsive materials [148]. After the scaffold is implanted, if there is no infection, the concentration of ROS in the microenvironment is extremely low, and the material remains stable. Once an infection occurs, the locally elevated ROS will specifically oxidize and break these sensitive bonds, thereby releasing the antibiotics, metal ions, or other antibacterial peptides that are pre-packaged within the material as needed [148].

To address the severe biofilm-induced hypoxia and ROS accumulation, Yang et al. integrated manganese oxide (MnO_2_) nano enzymes with catalytic activity similar to peroxidase into 3D printed scaffolds [150]. When in a microenvironment with high ROS content, MnO_2_ nano enzymes catalyze the decomposition of endogenous H_2_O_2_, converting it into oxygen (O_2_) and Mn^2+^ [150]. This continuous reaction not only can eliminate cytotoxic ROS and alleviate local hypoxia, thereby disintegrating the survival environment of anaerobic bacteria, but also actively promotes the processes of osteogenesis and angiogenesis [150].

#### 5.1.4. Enzyme-Responsive Strategies

Pathogenic bacteria secrete a large number of extracellular enzymes during colonization and the formation of biofilms, including hyaluronidase, lipase, β-galactosidase, or specific proteases, which can be used as stimulus factors to design enzyme-responsive systems [29]. The carrier matrix contains bonds that can be specifically cleaved by these enzymes [149]. As the matrix structure becomes loose, the antibacterial agents that were originally sealed inside the scaffold are released [149].

Zhang et al. linked matrix metalloproteinase (MMP)-cleaving peptides into a glycol network to form a MMP-responsive hydrogel, where localized ECM undergoes degeneration due to inflammation or mechanical injury; MMPs degrade this degenerated collagen and clear necrotic tissue, which exhibited strong adhesion and played an important role in M1 to M2 polarization, enhanced osteoblast differentiation, and promoted bone restoration [151]. Wall et al. developed enzyme-responsive porous nanofibrous scaffolds by co-electrospinning PCL with polyalanine (PAla) [152]. The incorporation of PAla enhanced the maximum tensile stress by 399% compared to PCL alone, while the scaffolds exhibited selective degradation of the PAla domain upon exposure to human neutrophil elastase (HNE)—a protease abundantly produced and overexpressed in chronic wound microenvironments, thus presenting a mechanically competent, wound-site-enzyme-responsive biomaterial for chronic wound healing applications [152]. Enzyme-responsive materials have great potential in achieving specific and selective antibacterial therapeutic treatments.

### 5.2. Exogenous Antibacterial Strategies

To overcome the limitations of passive release, exogenous-responsive strategies have been developed. By modulating stimulus parameters (intensity, duration, frequency), spatiotemporal precision control of antimicrobial efficacy can be achieved. These exogenous stimuli are primarily categorized into light, microwave, and ultrasound modalities [29]. Researchers have designed a range of materials capable of responding to these external stimuli to trigger therapeutic effects [153]. By leveraging the properties of biomaterials to generate ROS and heat *in vivo*, these approaches enable non-invasive and remotely controllable interventions for comprehensive antibacterial therapy and tissue regeneration [29].

#### 5.2.1. Light-Driven Strategies

The photoreactive antibacterial strategy mainly utilizes NIR irradiation. Compared to UV or visible light, NIR has relatively lower phototoxicity and better tissue penetration ability [154]. Its antibacterial mechanism mainly includes photothermal therapy (PTT) and photodynamic therapy (PDT) [155].

In PTT, photothermal materials absorb photon energy and convert it into local high heat energy that is released. This sudden temperature increase causes irreversible denaturation of bacterial proteins, impaired function of metabolic enzymes, and ultimately leads to bacterial death [156]. Xu et al. classified the PTT mechanism into three major categories: local plasma resonance (LSPR) effect, the generation and relaxation of electrons-hole pairs, and conjugation/hyperconjugation effects [157].

PDT essentially relies on photocatalytic action, generating electron-hole pairs through light excitation of semiconductor materials, which then drives the occurrence of redox reactions [158]. For these redox reactions to occur spontaneously, specific thermodynamic conditions must be met: the bottom of the conduction band (CB) must be more negative than the target reduction potential (*P*_red_), and the top of the valence band (VB) must be more positive than the target oxidation potential (*P*_ox_) [158]. Under specific wavelength irradiation, the excited photosensitizer transfers energy to surrounding O_2_ and water (H_2_O) molecules, generating highly cytotoxic ROS as intermediate products, including hydroxyl groups free radicals (•OH), superoxide free radicals ($•O_{2}^{-}$), hydrogen peroxide (H_2_O_2_), and singlet oxygen (^1^O_2_) [99,159].

**Figure 5:**
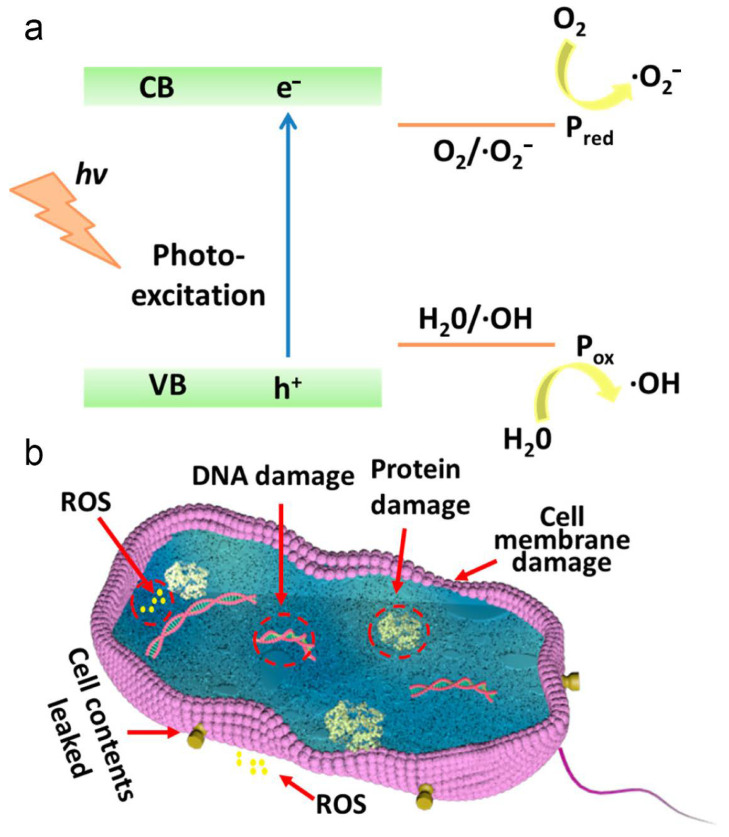
(**a**) Electronic structure of semiconductor photocatalysts changes in photocatalytic reactions; (**b**) Photocatalytic antibacterial mechanism diagram. Reproduced with permission from Ref. [161].

Taking TiO_2_ photocatalysis as an example (Figure 5a), when the incident photon energy exceeds its band gap, electrons jump from the VB to the CB, forming reactive electron-hole(e^−^-h^+^) pairs [160,161]. The positively charged holes in the VB interact with adsorbed hydroxyl ion (OH^−^)or H_2_O to produce highly active •OH, which can indiscriminately oxidize a broad spectrum of organic substances. Concurrently, the photogenerated electrons in the CB reduce dissolved O_2_ to yield ($•O_{2}^{-}$), further enriching the reactive radical pool [158].

The reaction process is shown in the following equations:

(1) TiO_2_ + hν → TiO_2_ (e^−^, h^+^)

(2) e^−^ + h^+^ → heat or hν

(3) h^+^ + OH^−^ → •OH

(4) h^+^ + H_2_O → •OH + H^+^

(5) $$e^{-}+O_{2}\;\to \;•O_{2}^{-}$$

Ultimately, the photocatalytic antibacterial process is driven by the severe oxidative damage inflicted by these ROS upon the pathogens [99]. As illustrated in Figure 5b, these ROS act through multiple mechanisms to breach bacterial defenses, penetrate the cell membrane, and oxidatively damage critical biomacromolecules such as proteins and DNA, thereby inducing the lethal leakage of intracellular substances and culminating in bacterial eradication [100].

**Figure 6:**
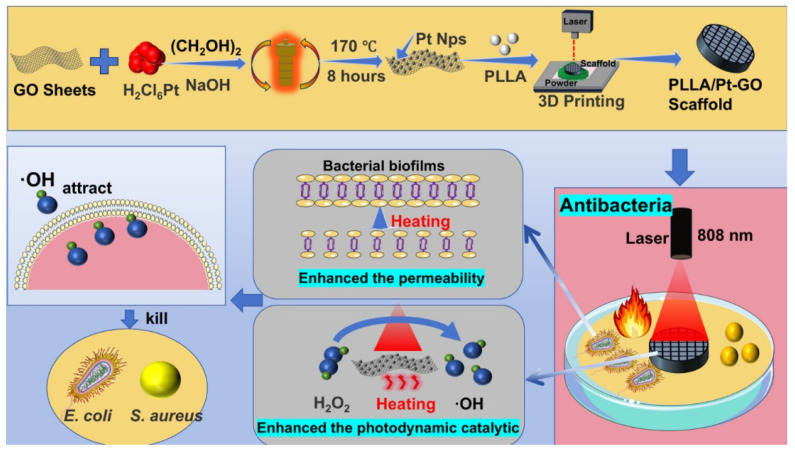
Schematic illustration of the fabrication process of the PLLA/Pt-GO scaffold and its underlying photodynamic-photothermal synergistic antibacterial mechanism. Reproduced with permission from Ref. [33].

Recent advancements have focused on developing heterogeneous nanomaterials that synergize both modalities to overcome the limitations of monotherapies. For instance, natural organic photosensitizers like curcumin have been integrated with plasmonic metals to enhance their stability and light-harvesting capabilities [162,163]. Liu et al. employed a photo–sono interfacial engineering strategy to fabricate a Cu sulfide (CuS)/curcumin hybrid, wherein an internal electric field promotes charge separation to enable rapid and synergistic antibacterial therapy via photothermal, photodynamic, and sonodynamic effects [163]. In the field of BTE, Liu et al. fabricated 3D printed forsterite scaffolds with a hydrothermal HA coating, wherein embedded free carbon enabled photothermal antibacterial activity while the HA layer promoted osteogenic differentiation without compromising mechanical integrity or photothermal performance [164]. Moreover, Shuai et al. constructed a laser 3D printed poly-l-lactic acid (PLLA)/platinum (Pt)–GO scaffold that leverages the photothermal effect of GO to disrupt bacterial biofilms and amplify the peroxidase-like photodynamic activity of Pt NPs, thereby achieving synergistic photothermal–photodynamic antibacterial efficacy against implant-associated pathogens (Figure 6) [33]. However, PTT often requires high localized temperatures (55–60 °C) that may cause collateral thermal damage to host tissues, and the limited tissue penetration depth of NIR light (a few millimeters) restricts its application for deep-seated orthopedic infections [34].

#### 5.2.2. Microwave-Driven Strategies

Microwave-responsive antibacterial utilizes microwaves as the external excitation source, with its electromagnetic wave characteristics falling between radio waves and infrared rays (typically operating at a frequency of approximately 2.45 GHz) [165]. Microwaves possess remarkable tissue penetration capabilities, with a penetration depth far exceeding that of NIR [166]. This property makes microwave-driven antibacterial materials suitable for treating deep bone infections, such as severe osteomyelitis [167]. The microwave-responsive antibacterial mechanism mainly involves microwave hyperthermia (MTT) and microwave dynamic therapy (MDT).

The mechanism of MTT lies in the intense interaction between the alternating electromagnetic field and the biological microenvironment and the dielectric components of the responsive scaffold [168]. When microwaves penetrate the tissue interface, they drive polar molecules (mainly H_2_O) and ions to perform high-frequency dipole rotation and translational oscillations at a frequency of billions of times per second [169]. This rapid and continuous molecular motion generates tremendous frictional force, whose kinetic energy is converted into local heat energy through the relaxation process and dissipated [169]. Additionally, the dielectric solid materials and ionic liquids in the biological matrix exhibit interface polarization and ionic conductivity properties, thereby significantly enhancing the photothermal conversion efficiency [165,169].

The implementation of MDT relies on the introduction of microwave sensitizing materials [168]. The dielectric heterogeneity of these sensitizing materials, when stimulated by microwaves, can generate high-density local hotspots [168]. These hotspots will form local strong electric fields, thereby causing the surrounding aqueous medium to ionize and release free electrons [168]. Subsequently, dissolved O_2_ captures these electrons, thereby initiating a series of chain reactions, ultimately leading to the generation of highly oxidizing ROS [35,168].

Jin et al. reported a molybdenum disulfide (MoS_2_)/carbon nanotube (CNT) heterojunction that enables combined MTT and MDT for treatment of *S. aureus*-induced chronic osteomyelitis [170]. Microwave irradiation simultaneously generates hyperthermia and ROS through enhanced charge accumulation and electron spillover at electromagnetic hot spots [170]. In the field of BTE, Zhan et al. primarily leveraged the MTT for antibacterial activity without exploring MDT [171], which reflects a broader limitation in current research.

#### 5.2.3. Ultrasound-Driven Strategies

Ultrasound, due to its outstanding tissue penetration ability and clinical safety, has become a highly promising exogenous regulatory method [34,172]. In the design of advanced biomaterials, the ultrasound-responsive antibacterial strategies mainly include two functional therapies: specifically utilizing the sonothermal effect and sonodynamic effect to achieve targeted bacterial elimination, while relying on the piezoelectric effect to promote bone tissue regeneration [173].

Ultrasound generates two types of effects: thermal effects and mechanical effects [174]. The thermal effect eradicates bacteria through localized hyperthermia induced by ultrasonic irradiation, whereas the mechanical effect serves as the key driving force underlying sonodynamic therapy (SDT) [174]. Initially, the sonothermal effect arises from the attenuation of ultrasound energy [175]. As ultrasound waves propagate through the tissues surrounding a stent, high-frequency mechanical vibrations generate localized friction and viscous resistance; this, in turn, triggers localized hyperthermia, thereby disrupting the structural integrity of robust bacterial biofilms, increasing cell membrane permeability, and ultimately leading to bacterial death [175].

**Figure 7:**
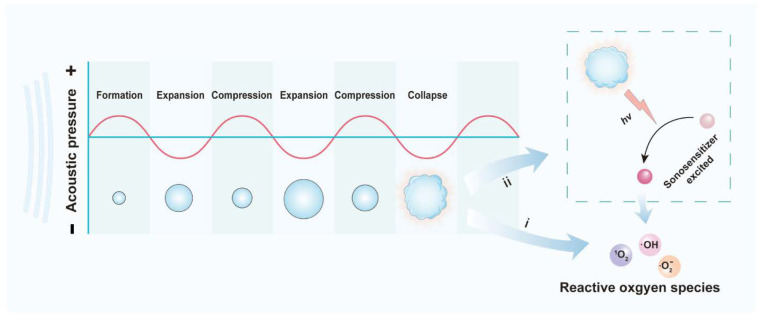
The popular mechanism of ROS generation under ultrasound irradiation. The cavitation bubbles undergo three stages of nucleation, bubble growth, and implosion under ultrasound irradiation, and then (i) generate ROS; (ii) release the energy to activate the sonosensitizer to generate ROS. Reproduced with permission from Ref. [104].

The core antibacterial effect of the ultrasound-responsive strategy stems from the sonodynamic effect, the underlying mechanism of which is driven by acoustic cavitation and sonoluminescence [104,176]. Under ultrasonic irradiation, microbubbles within the physiological fluid medium continuously form, expand, and violently collapse (Figure 7) [104]. This cavitation process generates severe mechanical shear forces that can physically disrupt biofilm [104]. The phenomenon of sonoluminescence arises as a secondary effect of cavitation: the violent collapse of these cavitation bubbles generates extreme local temperatures and pressures, thereby emitting transient light radiation [176]. This endogenous photon emission acts as an *in situ* excitation source for incorporated sonosensitizers, such as metal–organic frameworks (MOF) or semiconductor nanomaterials [176]. The excited sonosensitizers facilitate robust electron transfer and charge separation, culminating in the explosive generation of highly oxidative ROS [176]. Pan et al. developed an ultrasound-responsive MoS_2_ heterojunction scaffold that enhances bacterial membrane permeability and generates ROS via sonodynamic therapy, achieving 99% and 98.5% antibacterial efficacy against *S. aureus* and *E. coli* [177], respectively, as SDT-mediated ROS burst induces fatal oxidative stress, irreversibly destroying bacterial cellular structures and intracellular DNA to achieve thorough deep-tissue sterilization [178].

**Figure 8:**
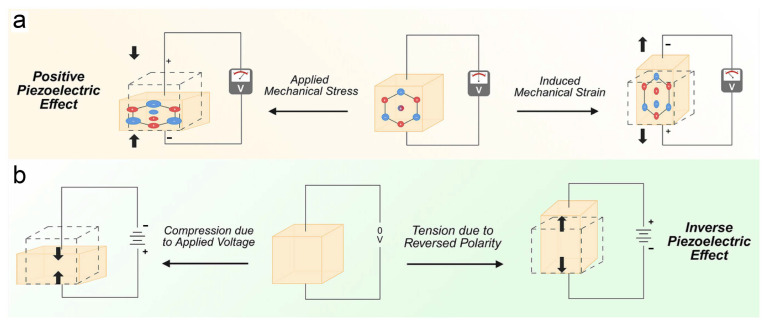
Characteristics of piezoelectric materials. (**a**) Schematic illustration of the positive piezoelectric effect; (**b**) Schematic illustration of the inverse piezoelectric effect. Reproduced with permission from Ref. [180].

Additionally, natural bone is intrinsically a biological piezoelectric material that generates microcurrents under physiological loading to regulate bone remodeling [18]. This behavior is due to what we refer to as the direct piezoelectric effect; applying mechanical force on the material (e.g., compression, vibration, ultrasound), which displace overlapping inner charge centers and generate surface charges [179]. On the other hand, there verse piezoelectric effect is caused by an external electric field that generates intrinsic polarization and a consequent change in the material’s shape (Figure 8) [180]. By incorporating ultrasound-responsive piezoelectric scaffolds, researchers can mimic this endogenous electro-mechanical microenvironment [181,182]. When exposed to ultrasonic mechanical vibrations, these responsive materials convert the mechanical energy of the ultrasound into localized electrical signals [173]. This ultrasound-driven electrical stimulation plays an important role in influencing surrounding osteoblasts. It significantly upregulates the expression of osteogenesis-related genes and activates osteogenic signaling pathways, thereby accelerating the differentiation of pre-osteoblasts [16]. Consequently, the synergistic interplay between the sonothermal and sonodynamic antibacterial effects and the piezoelectric stimulation of osteogenesis holds profound significance for the treatment of bone defects.

### 5.3. Multifunctional Smart Responsive Strategies

Due to the combined effects of trauma, congenital deformities, or tumor resection, bone defects often reach critical sizes, and these injuries often exceed the body’s own regenerative capacity [1]. Therefore, the clinical treatment of such defects needs to shift from the traditional single sterilization strategy to a multifunctional intelligent response strategy. These intelligent responsive antibacterial materials can simultaneously perform multiple therapeutic functions such as destroying biofilms and promoting osteogenesis, synergistically exerting antibacterial and promoting angiogenesis effects, or jointly eliminating infection and treating tumors.

**Figure 9:**
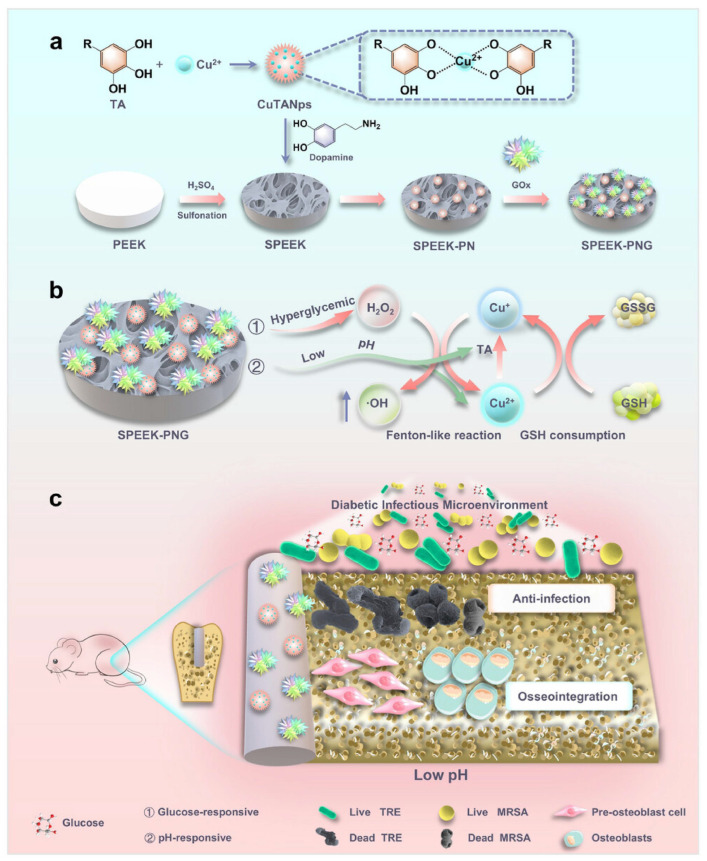
Schematic illustration of the glucose/pH dual-responsive PEEK implant for diabetic bone defects. (**a**) Fabrication process of the functionalized implant co-immobilized with CuTANps and GOx; (**b**) The endogenous cascade-amplified antibacterial mechanism, involving glucose depletion and a Cu-mediated Fenton-like reaction; (**c**) *In vivo* application demonstrating simultaneous infection eradication and osseointegration. Reproduced with permission from Ref. [31].

To achieve the dual functions of antibiofilm activity and osteogenesis, smart responsive materials leverage specific microenvironments or external stimuli to simultaneously disrupt bacterial defense mechanisms and promote osteogenic mineralization. Ding et al. developed a glucose/pH dual-responsive PEEK implant for diabetic osteomyelitis (Figure 9) [31]. By immobilizing glucose oxidase (GOx) and Cu-tannic acid nanoparticles (CuTANps) on the porous sulfonated PEEK surface (Figure 9a), GOx consumes endogenous glucose and, while effectively killing bacteria, converts it into gluconic acid and H_2_O_2_ [31]. The generated gluconic acid further reduces the local pH value, thereby triggering the dissociation of CuTANps and the strong release of Cu^2+^. After Cu^2+^ is reduced to Cu^+^, it accelerates the Fenton-like reaction with the locally produced H_2_O_2_, thereby generating •OH (Figure 9b) [31]. This endogenous stimulus response strategy not only achieves the clearance of resistant biofilms but also utilizes the synergistic effect between the released Cu ions and the porous structure, significantly enhancing the bone integration ability (Figure 9c) [31].

Successful reconstruction of severe bone defects relies not only on infection clearance and bone regeneration but, crucially, on robust angiogenesis to establish the vascular networks necessary for nutrient delivery and sustained tissue regeneration. Chen et al. utilized DLP 3D printing technology to fabricate a piezoelectric ceramic scaffold composed of BaTiO_3_ and HA modified by ZnO NPs under low-intensity pulsed ultrasound (LIPUS) stimulation, this scaffold demonstrated potent antibacterial activity alongside significant osteogenic and angiogenic potential, offering a promising comprehensive strategy for the repair of infected bone defects [121]. Under LIPUS stimulation, acoustic cavitation physically disrupts bacterial membranes [175], while piezoelectric microcurrents polarize macrophages toward an anti-inflammatory M2 phenotype, thereby upregulating the expression of both osteogenic and angiogenic factors [16].

Addressing the challenges of tumor recurrence from residual cells, infection, and severe bone defects following bone tumor surgery requires multifunctional, responsive implant materials. To this end, Zhao et al. developed a composite scaffold based on CS/hydroxypropyltrimethyl ammonium chloride chitosan (HC)/HA/BP and employed a multi-stage photothermal strategy [183]. Under NIR irradiation, an initial photothermal stimulus—raising the temperature to just below 50 °C—enabled simultaneous antitumor and antibacterial therapy, effectively eliminating 95% of osteosarcoma cells, 97% of *E. coli*, and 92% of *S. aureus*. Subsequently, a milder hyperthermic stimulus at approximately 42 °C accelerated bone repair by upregulating heat shock proteins, significantly enhancing the *in vivo* expression levels of osteogenesis-related genes [183].

Multifunctional smart-responsive 3D printed implants offer a highly promising, comprehensive clinical strategy capable of effectively controlling severe infections, combating bone tumors, and promoting vascularization and bone tissue regeneration to address complex pathological defects.

To provide a clearer comparison of different smart responsive strategies, their stimuli, functional materials, and comparative advantages/limitations are detailed in Table 3.

## 6. Prospects: AI-Assisted 3D Printing of Bone Scaffolds

Traditional additive manufacturing methods often face challenges when designing composite materials, such as suboptimal mechanical compatibility, a lack of personalized design capabilities, and demanding computational requirements [37]. By leveraging powerful computational capabilities to analyze complex biological and mechanical data, AI provides a robust framework for overcoming these limitations, thereby driving the optimization of therapeutic strategies and the development of next-generation bone regeneration solutions [184].

### 6.1. Workflow and Fundamentals of AI-Assisted 3D Printing of Bone Scaffolds

**Figure 10:**
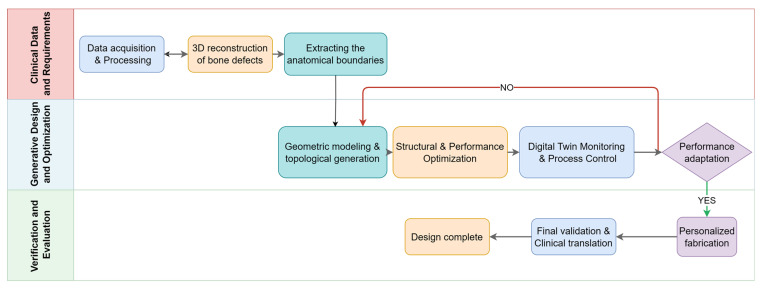
AI-Driven Bone Scaffold Fabrication Workflow Block Diagram.

In order to fully utilize AI technology in traditional additive manufacturing and overcome the challenges such as insufficient mechanical compatibility and high computational requirements, a systematic process must be established. This process typically consists of four key stages: data collection, model training, validation, and interpretability [185] (Figure 10).

**Figure 11:**
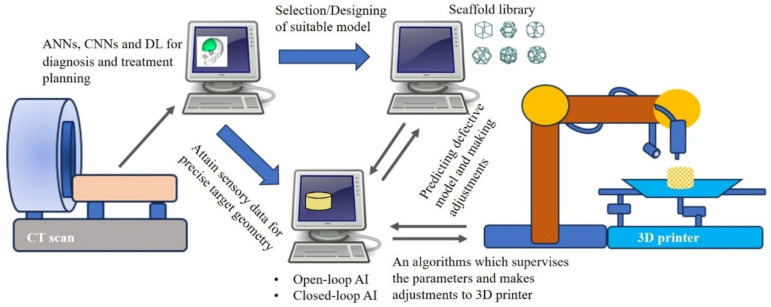
Schematic representation of a 3D scaffold fabrication network using AI. Reproduced with permission from Ref. [37].

Initially, high-quality datasets must be gathered. The data types mainly include patient-specific imaging data (computed tomography (CT)/magnetic resonance imaging (MRI) scans), material performance datasets (viscoelastic and mechanical parameters), and biological response data (cell proliferation and bone integration indicators) [185]. During the model training process, algorithms such as artificial neural networks (ANNs), convolutional neural networks (CNNs), and deep learning (DL) are employed to autonomously learn complex, nonlinear relationships within these diverse datasets, mapping structural designs to desired therapeutic outcomes (Figure 11) [186]. Subsequently, the model must undergo rigorous physical and experimental validation to prevent “overfitting,” ensuring they can accurately generalize predictions for new, unseen clinical scenarios rather than merely memorizing the training data [187]. Finally, interpretability remains crucial to address the opaque “black box” nature of DL and foster clinical trust, explainable AI (XAI) frameworks are increasingly utilized to interpret the underlying decision-making process [188]. This interpretability allows researchers to clearly understand how specific design parameters and feature weights influence the ultimate biomechanical and biological performance of the bone scaffold [188].

### 6.2. Applications of AI in 3D Printing

#### 6.2.1. Generative Design

The ability to rapidly and precisely optimize scaffold structures is a major advantage of AI-assisted 3D printing [189]. In recent years, generative design based on AI has made significant progress, enabling the automatic construction of novel and biomimetic porous structures, thus overcoming the limitations of traditional manual CAD design [190]. Guo et al. utilized a ML-driven multi-objective optimization framework to customize the design of a biological glass scaffold based on specific biomechanical adaptation requirements [190]. The researchers employed support vector regression (SVR) to establish a high-precision mapping between structure and performance [190]. Additionally, through DLP technology and combined with an inverse compensation strategy, high-fidelity printing was achieved, successfully controlling the manufacturing error of the theoretically optimized scaffold to below 3% [190].

#### 6.2.2. Scaffold Optimization

Beyond generating new structures, AI tools including ANNs, CNNs, and DL are capable of predicting mechanical properties, such as normalized effective elastic stiffness, without the need for time-consuming and complex iterative numerical simulations [186]. For instance, ANN-based optimization of Kagome structures has successfully balanced key design parameters like pore size, porosity, and strand diameter, resulting in a 43.2% increase in scaffold stiffness [186]. Furthermore, advanced predictive models, including the Hist Gradient Boosting Regressor, have demonstrated exceptional accuracy (up to 95.33%) in estimating the compressive strength of bone scaffolds prior to manufacturing, thereby significantly reducing the need for manual intervention and iterative trial and error processes [38].

#### 6.2.3. Digital Twins and Real-Time Process Control

In addition to the structural design, AI also significantly enhances the capabilities of process design and the development of biomaterials in the 3D printing process [191]. This improvement is largely attributed to the introduction of digital twin technology, which creates a dynamic virtual replica of the physical manufacturing environment. The manufacturing parameters of the biological printing system can be predicted, adjusted, and controlled autonomously in real time by AI, thereby reducing the error rate and ensuring repeatability [37]. This ability is crucial for eliminating minor physical disturbances, such as fluctuations in the diameter of the printed filament that may occur during extrusion molding [192]. At the same time, AI accelerates the innovation of composite biomaterials by optimizing surface modification techniques, thereby promoting cell proliferation and bone integration [193].

Ultimately, the integration of AI with 3D printing technology shows great application prospects in the field of personalized clinical translation [189]. AI can achieve the rapid preparation of scaffolds for specific patients, enabling them to precisely match the bone defect model, which is particularly beneficial for craniofacial reconstruction surgery and early hip-preserving treatment for femoral head necrosis [194]. Moreover, these models, which have been processed by AI algorithm models, can serve as reliable alternatives to preclinical animal experiments, effectively reducing research costs and minimizing immunological interference [37].

### 6.3. Limitations of AI in BTE

Although AI has great potential in optimizing 3D printed bone scaffolds, it is necessary to acknowledge that it also has several limitations. The primary challenge lies in the inherent “black box” nature of the algorithms [195]. Although these models can accurately predict mechanical properties and biological responses, the decision-making process behind them often lacks transparency, which brings serious trust and regulatory obstacles in clinical applications [195]. Additionally, AI models highly rely on the quantity and quality of the datasets, and these datasets related to bone defect patients are usually not easily accessible [195]. The scarcity of data may lead to overfitting or prediction bias of the models, thereby limiting the generalizability of AI-generated designs in different patient populations. Future research efforts must establish clear and standardized application guidelines to ensure that AI-driven methods are transparent, reproducible, and have a reliable verification basis, so that the research results obtained through computation can be transformed into clinical practice applications.

## 7. Clinical Translation and Challenges

### 7.1. Preclinical and Clinical Materials for Bone Scaffolds

The selection and development of biomaterials form the foundation of scaffold-driven bone regeneration. Materials must be rigorously evaluated across two distinct stages: preclinical and clinical.

During the preclinical stage, researchers focused on developing advanced composite bioinks. These materials typically consist of synthetic polymers, bioactive ceramics, and nanomaterials. These materials have been optimized to achieve excellent printing performance, degradation performance, and cell compatibility. Preclinical evaluations mainly rely on *in vitro* cell cultures and small animal models (such as critical-sized cranial defects in rats) to verify early osteogenic induction, vascularization, and structural integrity.

In contrast, the requirements for clinical materials are much more stringent than those for preclinical materials. To achieve true clinical transformation, biological materials must possess absolute repeatability and excellent long-term stability. Additionally, clinical materials must undergo a strictly standardized sterilization process without compromising their mechanical or biological properties, ensuring that they do not trigger an immune response upon implantation in the human body and are completely safe.

### 7.2. Critical Challenges in Future Clinical Translation

The primary challenge lies in the complex regulatory approval process. Currently, there is still a lack of standardized evaluation protocols for customized and individualized scaffolds, especially when using continuously evolving AI models to adjust the internal microstructure of individual patients.

Manufacturing clinical-grade bone scaffolds under strict current good manufacturing practice conditions requires a sterile laboratory environment, professional technicians, and expensive equipment. Achieving large-scale production while keeping production costs under control to support the treatment needs of a wide range of patients remains a significant economic obstacle.

Although short-term *in vitro* and *in vivo* studies have shown good cell attachment effects, ensuring long-term stable bone integration, preventing dynamic stress shielding, and the long-term immune response of patients are still difficult to predict.

## 8. Conclusions

In summary, the reconstruction of bone defects has significantly benefited from 3D printing technologies, specifically extrusion-based, photopolymerization-based, and powder-based methods. Personalized scaffolds featuring precisely controlled structural parameters can now be fabricated with high accuracy. Studies have demonstrated that maintaining an interconnected porosity within the range of 300–700 μm is effective in promoting bone ingrowth and nutrient transport. However, the issue of IAIs remains a pressing challenge that requires urgent resolution.

Although passive antimicrobial strategies offer a preliminary line of defense, they often struggle to achieve sustained release control and may harbor potential cytotoxicity. To address these limitations, smart responsive materials have been developed. These systems enable the active and controllable modulation of antimicrobial behavior in response to endogenous or exogenous stimuli, thereby facilitating on-demand therapy. Among exogenous stimulation methods, ultrasound-driven strategies demonstrate significant advantages in orthopedic applications. Unlike light-based therapies—which suffer from limited tissue penetration depth—ultrasound is capable of penetrating deep into tissues to treat deep-seated infections with minimal energy attenuation. Furthermore, while microwave irradiation can also achieve deep tissue penetration, its non-thermal antimicrobial mechanisms remain poorly understood, resulting in its relatively limited application in current bone scaffolds. In contrast, ultrasound can trigger the generation of ROS via sonodynamic therapy to eradicate biofilms. Simultaneously, ultrasound activates piezoelectric components within the scaffold to generate localized electrical signals. These microcurrents mimic the natural electromechanical environment of bone tissue, effectively stimulating osteoblast differentiation and angiogenesis. This dual-functional approach offers a more effective and reliable solution for treating infected bone defects.

Looking ahead, the integration of AI with 3D printing represents a pivotal step toward the fabrication of personalized bone scaffolds. AI enables the optimization of complex scaffold designs and the prediction of their mechanical and biological properties, thereby reducing the need for extensive empirical testing. Future research should focus on combining AI-driven structural optimization with exogenous-responsive materials; such advanced scaffolds would facilitate a more efficient translation from laboratory research to clinical practice, ultimately providing patients with safer and more reliable implants.

## Figures and Tables

**Table 1:** Comparison of advanced 3D printing technologies for bone scaffold fabrication.

Technology	Common Modalities	Typical Biomaterials	Advantages in BTE	Refs.
Extrusion	FDM DIW	PLA, PCL, PEEK, Hydrogels, Bioceramics	Broad material versatility; Cell viability and molecule bioactivity; Cost-effective	[42,43,44,45,46,47,48,49,50]
Photopolymerization	SLA DLP	HA, α-TCP, β-TCP, GelMA	Exceptional resolution; Remarkably smooth surface; Enables complex geometric architectures	[49,51,52,53]
Powder Bed Fusion	SLS EBM	Metal powder, Ceramics	High mechanical strength; Sintering preserves porous architecture	[54,55,56]

**Table 2:** Summary of Passive Antibacterial Strategies.

Classification	Antibacterial Agents	Antibacterial Mechanism	Effect	Refs.
Metal/Metallic Cations	Ag	Ion release	Antibacterial	[69,106]
	Cu	Ion release	Antibacterial, Angiogenesis	[111,113]
	Zn	Ion release	Antibacterial, Osteogenesis	[106,111]
	Ga	Ion release	Antibacterial, Anti-inflammatory, Angiogenesis	[116,117]
Metallic Oxides	ZnO	Ion release	Antibacterial, Osteogenesis	[121]
	TiO_2_	Photocatalysis	Antibacterial, Cytocompatibility	[125]
Organic Agents	Chitosan	Electrostatic interactions	Antibacterial, Cytocompatibility	[128]
	HACC	Electrostatic interactions	Antibacterial, Cytocompatibility	[101]
	AMPs	Membrane disruption	Antibacterial	[127]

**Table 3:** Summary of smart responsive antibacterial strategies.

Stimulus	Smart Responsive Materials	Antibacterial Mechanism	Advantage	Limitation	Refs.
pH	ZIF-8 mesoporous silica	Acid-triggered drug release	Self-activating	Relies on local physiological variations	[141,142]
Temperature	PNIPAM PU	Phase transition and drug burst release	Autonomous; Rapid localized release	Requires strict LCST tuning	[32,145]
Glucose	GOx-PEEK	Glucose starvation	Holistic microenvironment regulation	Dependent on metabolite levels	[31]
ROS	MnO_2_	Enzymatic ROS	Holistic microenvironment regulation	Dependent on metabolite levels	[150]
Enzyme	PAla	Enzyme-specific bond cleavage	Highly specific and selective release	Highly dependent on local specific enzyme concentration	[152]
Light	TiO_2_ CuS/Curcumin Pt-GO	PTT and PDT	Remote controllable	Limited penetration; Thermal damage risk	[33,160,163]
Microwave	MoS_2_/CNT	MTT and MDT	Deep tissue penetration	Less MDT application	[170]
Ultrasound	MoS_2_ BaTiO_3_	Cavitation effect and SDT	Exceptional penetration and piezoelectric osteogenesis	Requires sophisticated sonosensitizers	[121,177]

## Data Availability

The original contributions presented in the study are included in the article, further inquiries can be directed to the corresponding authors.
